# Acute Effect of Electronic Cigarette-Generated Aerosol From Flavored CBD-Containing Refill Solutions on Human Bronchial Epithelial Cells

**DOI:** 10.3389/fphys.2020.592321

**Published:** 2020-10-21

**Authors:** Noel J. Leigh, Maciej L. Goniewicz

**Affiliations:** Department of Health Behavior, Roswell Park Comprehensive Cancer Center, Buffalo, NY, United States

**Keywords:** electronic cigarettes, e-cigarettes, electronic nicotine delivery systems, flavorings, cannabinoids, inhalation, toxicity

## Abstract

**Introduction**: Although electronic cigarettes (e-cigarettes) were originally developed to deliver aerosolized nicotine to lungs, recent data have shown that consumers also use them for inhalation of other drugs, including cannabidiol (CBD). The aim of this study was to test the acute inhalation toxicity of flavored CBD-containing aerosols emitted from e-cigarettes.

**Methods**: Bronchial epithelial cells (H292) cells were exposed to aerosol generated from e-cigarettes refilled either with (1) propylene glycol solvent only (PG, control), (2) commercially purchased unflavored solution with CBD, or (3) commercially purchased solutions with and without CBD and with different flavors. The *in vitro* toxicological effects were assessed using the following methods: (1) trypan blue exclusion assay (cell viability), (2) neutral red uptake assay (metabolic activity), and (3) ELISA (concentrations of inflammatory mediators).

**Results**: Most flavored products with or without CBD were cytotoxic as compared to the air control. Overall, aerosols with CBD were more cytotoxic than aerosols without CBD irrelevant of the flavoring used in the product. Although, unflavored aerosols containing CBD in PG were significantly more cytotoxic than aerosols containing only PG, not all flavored products containing CBD were significantly more toxic than the same flavored products without CBD. Most CBD containing products significantly increase the concentration of cytokines released as compared to the same flavored products without CBD.

**Conclusion**: Different flavors show different cytotoxic effects in CBD-containing e-cigarettes. Aerosols emitted from CBD containing e-cigarettes were more cytotoxic than those emitted from CBD-free e-cigarettes.

## Introduction

Electronic cigarettes (e-cigarettes) are popular devices typically used to aerosolize flavored nicotine-containing solutions. The use of these devices for aerosolization for drugs other than nicotine, particularly cannabinoids like tetrahydrocannabinol (THC) and cannabidiol (CBD), has been gaining in popularity (Kenne et al., 2017; Trivers et al., 2019). Data from population-based studies have also indicated that a significant proportion of nicotine users also use cannabis (Lee et al., 2016; Trivers et al., 2019). While decriminalization of cannabis-derived products expands throughout individual states in the United States (Peace et al., 2016), products containing a mixture of cannabinoids are still classified as Schedule 1 substances under the United States Drug Enforcement Agency Controlled Substances Act. However, the Agriculture Improvement Act of 2018 allowed the promotion and marketing of products that only contain CBD without restrictions based on a claim that CBD-only products are derived from hemp, and not from cannabis. At this time, limited studies have been performed to investigate delivery and health effects of vaporized cannabinoids, including CBD.

Most commercially available CBD-containing e-cigarettes and refill solutions are available in wide array of flavors. Manufacturers of flavored CBD-containing products often use flavor descriptors that do not give a detailed depiction of the flavor profile present in the product, e.g., tobacco or cherry flavored. Additionally, flavor name on the container does not necessarily reflect the same flavor chemicals used between batches/manufactures. Finally, flavoring chemicals used in those products are not disclosed on packaging, bottles, or containers. This makes differentiation between CBD flavor types very difficult without smelling, tasting, or using analytical laboratory methods to distinguish between the flavor profiles.

We have previously successfully utilized the air-liquid interface (ALI) *in vitro* exposure models to study cellular effects of aerosols emitted from nicotine-containing e-cigarettes (Leigh et al., 2016). We reported that nicotine did not show significant cytotoxic or pro-inflammatory effects when delivered to bronchial epithelial cells with aerosols emitted from e-cigarettes. However, we found that flavorings used in nicotine-containing e-cigarettes significantly affected cytotoxicity of these products and induced inflammation. Cytotoxic effects of pure CBD as well as CBD oils have been observed in past studies in various cell lines (Cerretani et al., 2020; Urasaki et al., 2020). Additionally, in our pilot study (Leigh and Goniewicz, 2020), we showed that aerosols emitted from CBD-containing e-cigarettes may have cytotoxic effect and induce release of inflammatory markers in bronchial epithelial cells exposed *in vitro* to emissions from those products. Based on our preliminary findings, we hypothesized that flavorings used in CBD-containing e-cigarette products may affect the cytotoxicity of the aerosol and induce inflammatory response independently from CBD. Using our *in vitro* ALI exposure system, we exposed human bronchial cells to aerosols with or without CBD as well as with different flavors to evaluate cytotoxic and inflammatory responses.

## Materials and Methods

### Commercially Purchased E-Cigarette Device and CBD-Containing Flavored and Unflavored Refill Solutions

A puff-activated eGO tank (SmokeTek) was purchased online for this study. This product had a fixed battery output voltage of 3.8 V, and the coil in the CE4 tank had an average resistance of 4.0 Ω resulting in 3.6 W of power. We purchased one unflavored CBD-containing refill solution for e-cigarettes which was labeled CBD 1,000 mg/30 ml (33.3 mg/ml), Gentleman’s Brand. Additionally, five flavored CBD-free and CBD-containing refill solutions were also purchased online for use in this study: “Dark Side of the Moon” (Flavor 1, F1), “Midnight Express” (Flavor 2, F2), “Easy Rider” (Flavor 3, F3), “Lizard King” (Flavor 4, F4), and “Nice Dreams” (Flavor 5, F5), Cloud 9 Hemp. All flavored CBD-containing refill solutions had a labeled CBD concentration of 50 mg/30 ml (1.7 mg/ml). All commercially purchased refill solutions listed PG and VG as the solvent used, except the 1,000 mg/30 ml (33.3 mg/ml) unflavored CBD containing solution which listed polyethylene glycol (PEG) as the only solvent. All commercially purchased CBD-containing solutions were listed as industrial hemp derived and are not labeled as full spectrum. While the flavor classification of these solutions was unknown, we speculate that these products had a either a fruity, creamy, and buttery flavor or a chocolaty flavor based on their smell and GCMS profile of detected flavoring chemicals (Supplementary Table 2).

### Lab-Made Reference Refill Solutions

Unflavored solution containing 1.7 mg/ml CBD-only (PG + CBD), was prepared by diluting a commercially purchased unflavored CBD refill solution (33.3 mg/ml) with propylene glycol (PG, 99+% Acros Organics). Pure PG was also used as a solvent-only control during exposure (PG − CBD).

### GCMS Analysis of Flavored CBD-Containing Refill Solutions

Flavoring chemicals were identified in each tested refill solution with gas chromatography/mass spectrometry (GC/MS) method, as described previously (Leigh et al., 2016). GC/MS analysis showed that the primary cannabinoid in our products was CBD as listed on the packaging. Additionally, we found propylene glycol (PG) in all products as well as several flavoring compounds in the commercial products. Each product contained between 12 and 29 flavoring chemicals. Some flavoring chemicals, including benzyl alcohol, benzaldehyde, and piperonal were detected in more than one product; acetoin, 2,3-butanediol, and hydroxyacetone were detected in all products tested. The detailed list of detected flavoring chemicals and their sensory properties are provided in Supplementary Table 2. CBD concentrations were compared with the same peak area of analyzed samples. All commercially purchased CBD solutions did not contained delta-9 tetrahydrocannabinol (THC) as confirmed by GC/MS analysis.

### Generation of E-Cigarette Aerosols

Aerosol from the eGO e-cigarette device was generated using a Borgwaldt LX-1 (Richmond, VA, United States) single-port piston-operated smoking machine. The Health Canada Intense (HCI) puffing protocol was utilized with the following conditions: 2 s puff duration, every 30 s, with a 55-ml puff volume. The puffing protocol was used continuously for 55 puffs or 30 min following protocol described previously (Leigh et al., 2016). Thirty minutes was utilized as this was the minimum exposure time examined in which we saw significant differences between ENDS aerosol and the air control (data not shown). The CE4 tanks used in this study were re-filled to capacity (1.5 ml) 30 min before exposure for each condition. Each tank with refill solution was weighted before and after each run to determine if similar aerosol was exposed to H292 cells (Supplementary Table 3). Air only exposures (air control) were run during each experiment.

### Cell Exposure Conditions

The NCI-H292 bronchial epithelial cell line (ATCC) was used for all experimental conditions. Cells were exposed directly to freshly generated aerosol in an ALI as described previously (Leigh et al., 2016). During cell exposure to air or e-cigarettes aerosol, fresh media were cycled over the basal side of the permeable support at a flow rate of 5 ml/min.

### Toxicity Assays

Metabolic activity of exposed H292 cells was measured by neutral red uptake assay as described previously (Leigh et al., 2016). Cell viability was measured by trypan blue assay as described previously (Leigh et al., 2016). Six cytokines (IL-1β, IL-6, IL-10, CXCL1, CXCL2, and CXCL10) were measured as markers of cell inflammatory response using commercially available ELISA kits (CXCL2 Abcam, all others R&D Systems). For all assays, the manufacturer’s protocols were followed. ELISA results are presented as concentration divided by the number of live cells determined with the trypan blue assay.

### Statistical Analysis

Statistical analysis was performed using Prism version 8.4.2 (GraphPad). Kruskal-Wallis non-parametric tests with an uncorrected Dunn’s multiple comparison test were performed for each study outcome to compare: (1) the mean rank of tested refill solution vs. air control and (2) the mean rank of tested refill solution vs. PG-only solvent control. A Mann-Whitney *t*-test was performed for each study outcome to compare the statistical difference between PG-based refill solutions with and without CBD. Mann-Whitney *t*-tests were also used to compare each flavored tested solution with and without CBD for each study outcome. All experiments were performed in at least triplicate, with each outcome measured three times per experiment.

## Results

### Cytotoxic and Pro-inflammatory Effects of Exposure to Aerosols Generated From Unflavored CBD-Containing Refill Solutions (Effect of CBD)

**Figure 1:**
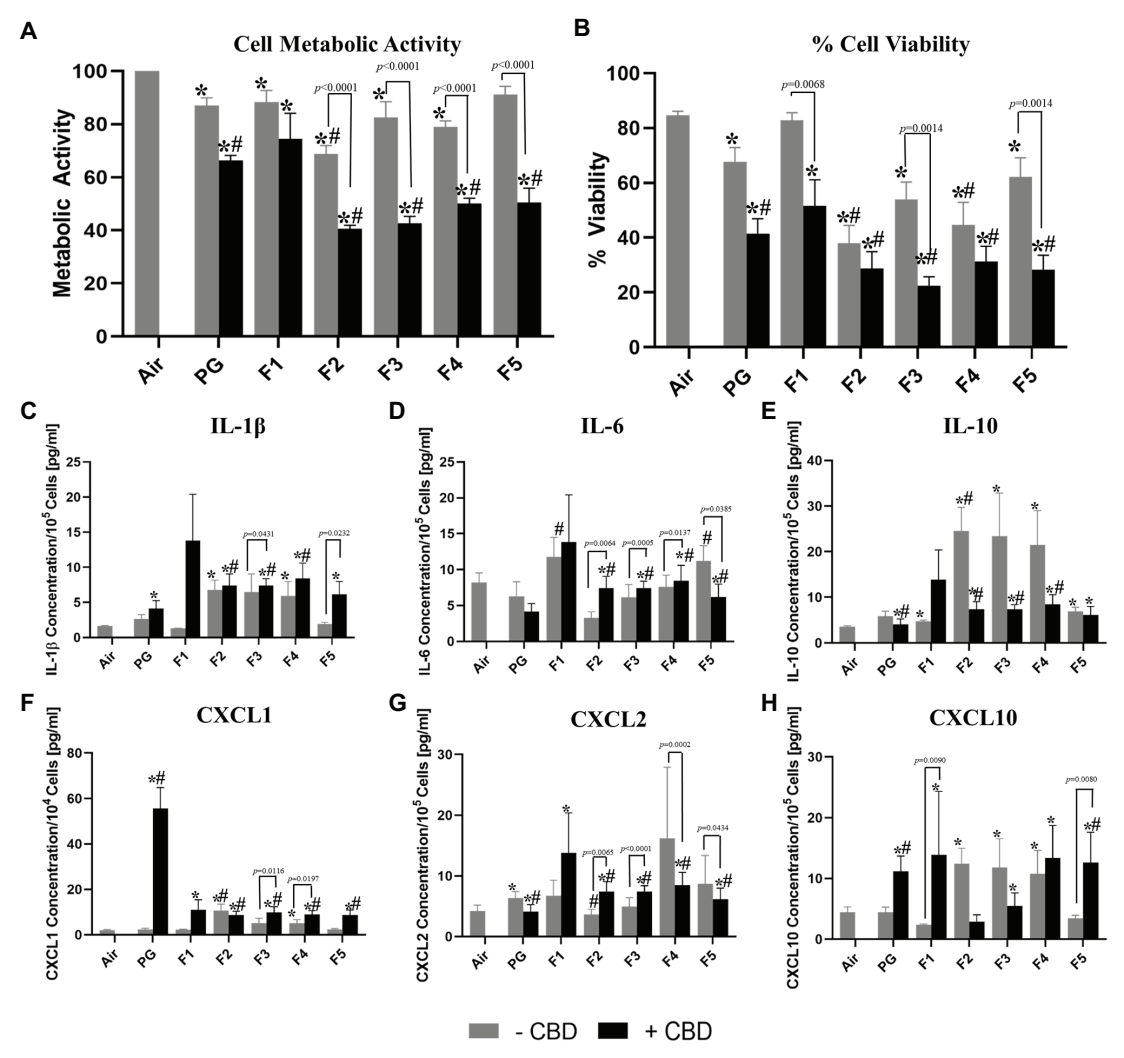
Comparison of cellular toxicity **(A,B)** and levels of released inflammatory mediators (cytokines/myokine, **C-H**) from H292 bronchial epithelial cells directly exposed at the air-liquid interface to 55 puffs of flavored and unflavored cannabidiol (CBD)-containing and CBD-free aerosols. All aerosols were generated from an eGO tank system, with battery output voltage set to 3.8 V and refilled with propylene glycol (PG)-only solution with the same CBD concentrations (1.7 mg/ml). Flavored refill solutions include: “Dark Side of the Moon” (Flavor 1, F1), “Midnight Express” (Flavor 2, F2), “Easy Rider” (Flavor 3, F3), “Lizard King” (Flavor 4, F4), and “Nice Dreams” (Flavor 5, F5) ^*^Indicates significant difference from the air control and ^#^indicates significant difference from the PG only solvent control (*p* < 0.05; Kruskal-Wallis test). Values are mean ± SEM. Results for Cell Metabolic Activity **(A)** were normalized to the air control.

Aerosols generated from unflavored CBD-containing solution (PG + CBD) was found to be significantly more cytotoxic on bronchial epithelial cells than aerosols from unflavored CBD-free solution (PG − CBD; *p* < 0.0024) for both cytotoxicity assays (Figure 1). When examining the inflammatory mediators, we observed a small but statically significant decrease in the anti-inflammatory cytokine IL-10 (*p* = 0.0442) and the pro-inflammatory cytokine CXCL2 (*p* = 0.0400) after exposure to PG + CBD compared to PG − CBD (Figures 1E,G). Additionally, we detected a significant increase in the pro-inflammatory cytokines CXCL1 (*p* = 0.0010) and CXCL10 (*p* = 0.0288) after exposure to PG + CBD compared to PG − CBD (Figures 1F,H).

### Cytotoxic and Pro-inflammatory Effects of Exposure to Aerosols Generated From Flavored CBD-Free Refill Solutions (Effect of Flavors)

Aerosol generated from various flavored CBD-free solutions (F1, F2, F3, F4, and F5) differed significantly in their toxicity of bronchial epithelial cells (Figure 1). Cell viability and metabolic activity of H292 cells decreased significantly (*p* < 0.0252) as compared to air after exposure to all flavored aerosols without CBD except; F5 − CBD for metabolic activity and F1 − CBD for percent cell viability (Figures 1A,B). F2 − CBD was found to be significantly different from the PG solvent control (PG − CBD) for metabolic activity (*p* = 0.006) and % cell viability (*p* = 0.0043, Figures 1A,B). F4 − CBD was also found to be significantly different from the PG − CBD for % cell viability only (*p* = 0.0371, Figure 1B).

When examining the ELISA results, we found significant differences between all five tested flavors without CBD and the air control for IL-10 (*p* < 0.0365, Figure 1E), all flavors except F1 − CBD and F5 − CBD for CXCL10 (*p* < 0.0361, Figure 1H) as well as only F2 − CBD and F4 − CBD for IL-1β and CXCL1 (*p* < 0.0341, *p* < 0.0313, respectively, Figures 1C,F). When comparing results from exposure to aerosols generated from the PG-only control solutions (PG − CBD) and exposure to aerosols generated from flavored CBD-free refill solutions, we observed that flavor F1 − CBD generated higher levels of IL-6 (*p* < 0.0172, Figure 1D), as well as flavor F2 − CBD generated higher levels of CXLC2 (*p* < 0.0444, Figure 1G). While only F2 − CBD generated lowers levels of IL-10 and CXCL1 (*p* = 0.0050 and 0.0012, respectively, Figures 1E,F) and exposure to F5 − CBD resulted in decreased levels of IL-6 (*p* = 0.0172, Figure 1D).

### Cytotoxic and Pro-inflammatory Effects of Exposure to Aerosols Generated From Flavored CBD-Containing Refill Solutions (Cumulative Effect of CBD and Flavors)

Cell viability and metabolic activity of H292 cells decreased significantly (*p* < 0.0009) as compared to air control after exposure to all flavored aerosols with CBD (Figures 1A,B). All flavors except F1 were significant different than the PG-only solvent control for both cytotoxicity measures (*p* < 0.0099, Figures 1A,B).

When examining the ELISA results, we found that exposure to all five flavors with CBD resulted in significantly higher release of CXCL1, CXCL2, and CXCL10 (*p* < 0.0349, Figures 1F–H), except for F2 + CBD for CXCL10, than exposure to the air control. Additionally, all flavors with CBD increased release of F1 + CBD for IL-1β, IL-6, and IL-10 (*p* < 0.0066, Figures 1C–E) as compared to air controls. When comparing flavored refill solutions with CBD to the PG-only control (PG − CBD), we observed significant differences between all five flavors except F1 for IL-6, CXCL1 and CXCL2 (*p* < 0.0234, Figures 1D,F,G), F2–F4 for IL-1β, and IL-10 (*p* < 0.0222, Figures 1C,E), as well as IL-1β and IL-10 (*p* < 0.0279, Figures 1C,E). Exposure to F5 + CBD resulted in higher release of CXCL10 (*p* = 0.0467, Figure 1H) compared to PG-only control.

All flavored refill solutions with CBD were significantly different from the unflavored + CBD control except F1 for metabolic activity (*p* < 0.0298) and IL-6 (*p* < 0.0337) as well as for only flavor F3 for % cell viability (*p* = 0.0355) and IL-1β (*p* = 0.0277, Supplementary Table 1).

All flavored refill solutions without CBD were found to be significantly different from those with CBD (*p* < 0.0434) except flavor F1 for metabolic activity, IL-6 and CXCL2 (Supplementary Table 1). CBD-containing flavors F1 and F5 (F1 + CBD and F5 + CBD) showed significantly stronger effects than the same flavors without CBD (F1 − CBD and F5 − CBD) for % cell viability and CXCL10 (*p* < 0.0068 and *p* < 0.0090, Supplementary Table 1). CBD-containing flavor F3 (F3 + CBD) showed significantly stronger responses compared to CBD-free flavor F3 (F3 − CBD) for % cell viability, IL-1β and CXCL1 (*p* < 0.0431, Supplementary Table 1). Exposure to flavor F4 with CBD (F4 + CBD) resulted in a significant increase of the concentration of CXCL1 (*p* = 0.0197) as compared to the same flavor without CBD (F4 − CBD, Supplementary Table 1).

## Discussion

We presented novel findings on the cytotoxic effects of flavored aerosols emitted from CBD-containing e-cigarettes. Our study aimed to examine the acute *in vitro* effects of several commercially available products using an established ALI model. Consistent with our pilot study (Leigh and Goniewicz, 2020), we confirmed that CBD when vaporized with e-cigarette refill solutions (PG + CBD) shows the cytotoxic effects on bronchial epithelial cells (Figure 1). Additionally, we found that PG + CBD refill solutions resulted in increased release of several pro-inflammatory cytokines, including IL-1β, CXCL1, and CXCL10 (Figure 1). These results are consistent with other CBD studies showing increase cytotoxicity and suppression of viability of cells exposure to CBD (Cerretani et al., 2020; Urasaki et al., 2020). These results are important at a time when products containing CBD are being widely marketed as goods with potential health benefits. While the majority of the CBD containing products on the market are sold as a tincture or edibles to be taken orally or used topically with limited scientific research justifying their use, we have shown that these products may have potential adverse respiratory effects when inhaled as e-cigarette aerosols.

Consistent with findings from our study examining *in vitro* effects of flavored nicotine-containing refill solutions (Leigh et al., 2016), different flavors in CBD-containing products showed different cytotoxic effects. We presented novel findings showing that in each of the five examined flavors without CBD, there are significant differences from air control in the measured cytotoxicity assays (Figure 1). Importantly, the PG-only solvent control also showed increased cytotoxicity compared to the air control (Figure 1), suggesting that solvents used in e-cigarettes may also independently contribute to the cytotoxic effects of the aerosols emitted from those devices.

An important finding from our study is that some commercially purchased products were more cytotoxic than others. This is likely a consequence of the differing flavoring chemical present in the various refill solutions. While the exact flavoring compound(s) responsible for this increase cytotoxicity was not determine in this study, we have noticed that some flavoring chemicals were only present in those flavored products that showed increased cytotoxicity, such as ligustrazin in F2 (Supplementary Table 2). Additionally, new compounds may be created when flavored refill solutions are heated inside e-cigarette devices. There is a need for further research identifying which flavoring chemicals are responsible for increasing cytotoxicity in flavored refill solutions. Identification of such highly cytotoxic flavoring chemicals may inform development of product standards and future products regulation to assure consumer safety.

Another important result of our study is the observed cumulative effect of flavorings and CBD present in all tested refill solutions. We observed a significant increase in cytotoxicity for all flavors as well as increased release of pro-inflammatory cytokines for most flavors, when comparing flavored refill solutions with and without CBD (Figure 1). Flavored refill solutions with CBD also resulted in a significant increase in cytotoxicity and production of pro-inflammatory mediators as compared to the unflavored controls containing only CBD (PG + CBD, Figure 1). Importantly, we did not observe similar effects for nicotine in our previous study (Leigh et al., 2016) since addition of nicotine to PG only solutions did not significantly affect the toxicity of the aerosol from previously tested refill solution. In contrast to nicotine, addition of CBD to flavored refill solutions amplify the observed biological responses. However, it should be noted that aerosol characteristics are highly linked to the device used, if other devices were used these results may not necessarily be the same.

In this study, we measured several pro- and anti-inflammatory mediators and found a significant increase in pro-inflammatory mediators IL-1β, CXCL1, CXCL2, and CXCL10 as well as a significant decrease in anti-inflammatory mediator IL-10 when H292 cells were exposed to flavored CBD refill solutions. While determining the mechanism behind these pro- and anti-inflammatory cytokines/chemokines was not our primary hypotheses, we believe the increase inflammation observed may be a result of co-administration of the solvent, propylene glycol, commonly used in refill solutions along with CBD. These results differ from that of (Urasaki et al., 2020) that found CBD was only cytotoxic in its pure form and not when diluted in oil. However, in our study, we used a different solvent which may explain these results. One of the important limitations of our study is that even though we purchased products of the same brand with and without CBD from the same supplier, we found some differences in flavoring composition of those products (Supplementary Table 2). This indicates that either there were additional additives present in CBD-containing refill solutions, that there may have been some inconsistencies between batches or that the flavor name present on the packaging does not necessarily use the same flavoring chemicals consistently to make that flavor profile. It should be noted that all commercial refill solutions were only qualitatively analyzed for flavoring chemicals. Future quantitive studies should be conducted to verify the presents and concentration of each of these flavoring compounds. Additionally, dose-responses experiments may be needed to precisely measure potential toxic effects of those products.

Another limitation of this study is that only one commercial product, in one concentration was used as the unflavored CBD containing refill solutions. Future studies should examine multiple unflavored CBD products, in multiple concentrations to determine if cytotoxic effects observed in our study are dose dependent. Although we tested five different flavored refill solutions with and without CBD, all products selected for our study came from a single manufacturer. Additional research should examine a large number of flavored CBD containing refill solutions from multiple manufacturers to verify our findings. Finally, our study only used a single physiologically relevant cell line to examining cytotoxicity and inflammatory endpoints. This culture model does not mimic the pseudostratified columnar epithelial cells of the human bronchial epithelial tissue comprising of goblet, cilia, and basal cells. While this method is useful for surveying acute effects of inhaled mixtures, it may not necessarily correlate to the respiratory effects seen with long-term use of these products in humans. Future studies using 3D culture and observational human trials would be useful to further verify these studies and understand the mechanisms involved.

A final limitation of this study is that no quantitative measurements of dosimetry were performed other than observed weight of refill solutions before and after use (Supplementary Table 3). With these measurements, we observed that the eGO e-cigarette with the CE4 tank had inconsistent delivery between runs with on average more aerosol delivered to the trials with refill solutions not containing CBD. We would expect the observed effects for CBD containing refill solutions would be further increased if similar amounts of aerosol were delivered to these trials. Future studies should include measurements of physical parameters such as contact angle, surface tension, and viscosity as well as other physical measurement such as quantitative chemical analysis and pH of refill solutions. Additionally, identification of other physical changes in the exposure system including pH and osmotic concentration would be useful to eliminate these as possible causes of observed results. The osmotic concentration of pure PG is upwards of 13,000 mOsm. Normal osmotic concentration for most vertebrate cells range from 260 to 320 mOSM/kg (Ian, 2000). As a result, it is possible some of the effects observed in this study are a result of a difference in osmotic concentration as demonstrated previous studies (Zhang et al., 2019).

In summary, the results of our *in vitro* study suggest potential harmful respiratory effects of flavorings and CBD when inhaled simultaneously with aerosols emitted from e-cigarettes. As use of cannabis-derived vaping products are increasing, studies are urgently needed to evaluate potential health consequences in users of these substances, particularly respiratory effects on chronic inhalation of flavored CBD-containing vaping products.

## Data Availability Statement

The raw data supporting the conclusions of this article will be made available by the authors, without undue reservation.

## Author Contributions

MG and NL contributed to the conception of the work and data analysis, and drafted the manuscript. NL ran experiments. Both the authors approved the final version of the manuscript. MG has full access to all study data and takes responsibility for the integrity of the data and accuracy of the data analysis.

### Conflict of Interest

MG reports grants from Pfizer and served as a scientific advisory board member to Johnson & Johnson, pharmaceutical companies that manufacture smoking cessation drugs. The remaining author declares that the research was conducted in the absence of any commercial or financial relationships that could be construed as a potential conflict of interest.

## Supplementary Material

Supplementary Table 1 Unpaired nonparametric Mann-Whitney comparisons between the same commercially manufactured flavored refill solutions with and without cannabidiol (CBD), 1.7 mg/ml. Flavored refill solutions with CBD were also compared to unflavored refill solutions without CBD, 1.7 mg/ml. Values are mean ± SD.

Supplementary Table 2 Qualitative comparison of commercially purchased flavored electronic cigarette (e-cigarette) refill solutions with and without CBD, 1.7 mg/ml, using gas chromatography. Flavored refill solutions include: “Dark Side of the Moon” (Flavor 1, F1), “Midnight Express” (Flavor 2, F2), “Easy Rider” (Flavor 3, F3), “Lizard King” (Flavor 4, F4), and “Nice Dreams” (Flavor 5, F5). Qualitative detection of a compound is indicated with an X when identified in both National Institute of Standards and Technology (NIST) and Mass Spectra of Flavors and Fragrances of Natural and Synthetic Compounds (FFNSC) mass spectrometry libraries.

Supplementary Table 3 Average mass (mg) of refill solution used per trial. Flavored refill solutions include: “Dark Side of the Moon” (Flavor 1, F1), “Midnight Express” (Flavor 2, F2), “Easy Rider” (Flavor 3, F3), “Lizard King” (Flavor 4, F4), and “Nice Dreams” (Flavor 5, F5). Values are mean ± SD.
